# Whole-Genome-Based Survey for Polyphyletic Serovars of *Salmonella enterica* subsp. *enterica* Provides New Insights into Public Health Surveillance

**DOI:** 10.3390/ijms21155226

**Published:** 2020-07-23

**Authors:** Zhiqiu Yin, Jiaheng Liu, Binghai Du, Hai-Hua Ruan, Yi-Xin Huo, Yuhui Du, Jianjun Qiao

**Affiliations:** 1 Key Laboratory of Molecular Medicine and Biotherapy, School of Life Science, Beijing Institute of Technology, Beijing 100081, China; yzq7873728@126.com; 2 College of Life Science, Nankai University, Tianjin 300350, China; 3 Shandong Engineering Research Center of Plant-Microbial Restoration for Saline-Alkali Land, College of Life Sciences, Shandong Agricultural University, Tai’an 271000, China; bhdu@sdau.edu.cn; 4 Key Laboratory of Systems Bioengineering (Ministry of Education), School of Chemical Engineering and Technology, Tianjin University, Tianjin 300072, China; liujiaheng929@126.com; 5 SynBio Research Platform, Collaborative Innovation Centre of Chemical Science and Engineering (Tianjin), Tianjin University, Tianjin 300072, China; 6 Tianjin Key Laboratory of Food Science and Biotechnology, College of Biotechnology and Food Science, Tianjin University of Commerce, Tianjin 300134, China; ruanhaihua@tjcu.edu.cn

**Keywords:** *Salmonella*, serotyping, polyphyletic serovar, niche-specific adaptation, pathogenicity, antimicrobial resistance, public health surveillance

## Abstract

Serotyping has traditionally been considered the basis for surveillance of *Salmonella*, but it cannot distinguish distinct lineages sharing the same serovar that vary in host range, pathogenicity and epidemiology. However, polyphyletic serovars have not been extensively investigated. Public health microbiology is currently being transformed by whole-genome sequencing (WGS) data, which promote the lineage determination using a more powerful and accurate technique than serotyping. The focus in this study is to survey and analyze putative polyphyletic serovars. The multi-locus sequence typing (MLST) phylogenetic analysis identified four putative polyphyletic serovars, namely, Montevideo, Bareilly, Saintpaul, and Muenchen. Whole-genome-based phylogeny and population structure highlighted the polyphyletic nature of Bareilly and Saintpaul and the multi-lineage nature of Montevideo and Muenchen. The population of these serovars was defined by extensive genetic diversity, the open pan genome and the small core genome. Source niche metadata revealed putative existence of lineage-specific niche adaptation (host-preference and environmental-preference), exhibited by lineage-specific genomic contents associated with metabolism and transport. Meanwhile, differences in genetic profiles relating to virulence and antimicrobial resistance within each lineage may contribute to pathogenicity and epidemiology. The results also showed that recombination events occurring at the H1-antigen loci may be an important reason for polyphyly. The results presented here provide the genomic basis of simple, rapid, and accurate identification of phylogenetic lineages of these serovars, which could have important implications for public health.

## 1. Introduction

The *Salmonella enterica* subspecies *enterica* is one of the most important bacterial enteric pathogens worldwide and is the main causative agent of typhoid fever, paratyphoid fever, and the foodborne illness salmonellosis in humans and other warm-blooded animals [1]. Globally, *S. enterica* has resulted in the highest medical burden, causing an average of 4.07 million disability adjusted life years between 1990 and 2012 [2]. Salmonellae are identified by Kauffmann–White–Le Minor serotyping and include more than 2600 serovars, 1500 of which fall within *S. enterica* subspecies *enterica* [3]. Approximately 50 serovars account for 99% of all clinical isolates of *Salmonella* from humans and domestic mammals, and all of these 50 serovars belong to the subspecies *enterica* [4]. Traditional serotyping has been used for classification, identification, and epidemiological investigation due to its user-friendly design. As with most typing methods, whether this typing method based on antigenic formulas reflects accurate evolutionary relatedness is always a question in the field. Serovars have often been shown to be correlated with host range and disease, while the host and/or disease variety within an individual serovar needs to be further subdivided by genetically informative methods.

When all strains of an individual serovar share a recent common ancestor and form a single lineage in a phylogenetic tree, the serovar can therefore be considered monophyletic. For example, Typhimurium and Typhi [5,6,7], among others, appear to be monophyletic. However, many serovars are polyphyletic, containing multiple lineages that do not share a most recent common ancestor. For example, Newport is polyphyletic, showing a high level of genomic diversity and at least three lineages associated with distinct geographical regions and hosts [8,9,10]. Paratyphi B is also polyphyletic and can cause diseases ranging from self-limiting gastroenteritis to severe systemic infections. Despite the considerable predicted diversity of Paratyphi B, there remain few categorization methods that subdivide the strains into lineages that are congruent with the disease phenotypes of the strains. A few pioneering studies have shown the polyphyletic nature of Paratyphi B, Kentucky, and Newport at serovar-level resolution using whole-genome sequencing (WGS) data [11,12,13]. For example, Connor et al. used WGS data to reconstruct phylogeny and population structure, and the phylogenetic discussion focused on distinct lineages with various abilities to cause invasive disease. Based upon their analysis, the specific Paratyphi B lineage-PG1 is significantly associated with invasive disease [11]. In these cases, the strains in polyphyletic serovars confound epidemiological investigations because serotyping cannot correctly identify the genetic differentiation of these strains. More importantly, Newport and Paratyphi B are consistently ranked in the U.S. Centers for Disease Control and Prevention (CDC)’s list of the top 20 *Salmonella* serovars attributed to human illness. Thus, accurate subtyping and subsequent clustering of isolates associated with diseases and/or outbreaks is essential for successful investigation and epidemic tracing. However, putative polyphyletic serovars have not yet been comprehensively identified and further analyzed systematically based on public WGS-data.

In recent years, with decreasing costs and increasing feasibility of next-generation sequencing, large amounts of genomic data have been generated. There are more than 5000 *Salmonella enterica* genomes available in the National Center for Biotechnology Information (NCBI) GenBank up to March, 2017. Furthermore, the EnteroBase database has currently deposited more than 200,000 *Salmonella* genomes [14]. WGS offers a tremendous advantage over other pathogen-typing methods, as this method offers a standardized universal solution for high-resolution typing [15]. Phylogeny and population structure analysis based on whole-genome data provide us with the capacity to identify the evolutionary diversity and quantify the divergence between lineages.

In this study, 4498 genomes of *S. enterica* subsp. *enterica* strains in NCBI GenBank were collected and screened to identify the putative polyphyletic serovars based on the global multi-locus sequence typing (MLST) phylogeny. Four serovars, Montevideo, Bareilly, Saintpaul, and Muenchen, were further investigated by a fine-scaled, accurate phylogeny and population structure based on WGS data. The putative lineage-specific niche preference was exploited. To expand the understanding of divergence and adaptation to specific niches, core genome, pan genome and lineage-specific genomic contents were characterized. The different characteristics (e.g., metabolic modules, *Salmonella* pathogenicity islands (SPIs), prophages, fimbrial operons, effectors, and antimicrobial resistances) occurred in distinct lineages were investigated to reveal the potential divergences in pathogenicity and epidemiology within serovar. Furthermore, the CRISPR-Cas system as an indicator of genomic diversity in the polyphyletic and multi-lineage serovars will be evaluated.

## 2. Results and Discussion

### 2.1. Identification of Putative Polyphyletic Serovars Based on MLST Phylogeny

**Figure 1:**
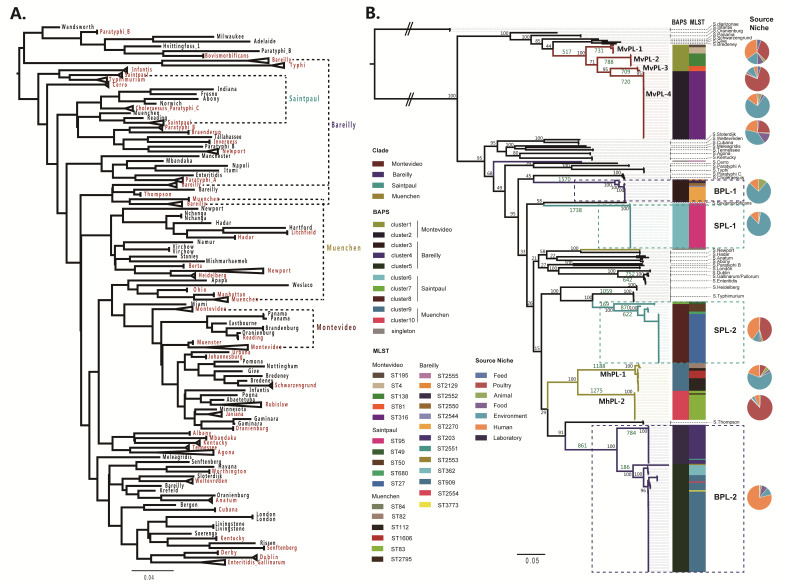
Phylogenetic analysis of the *Salmonella* strains. (**A**) Maximum-likelihood (ML) tree based on the 7 multi-locus sequence typing (MLST) genes among 4498 strains. Clade labels shown in red color clustered with more than 3 strains. The clusters of putative polyphyletic serovars marked with dotted lines. (**B**) Core genome phylogenetic analysis of 347 strains. Serovars Montevideo, Bareilly, Saintpaul, and Muenchen were designated as MvPL, BPL, SPL, and MhPL, respectively. The black values of the primary nodes of the tree are the bootstrap values (100 replicates). The single nucleotide polymorphisms (SNPs) on important branches are also shown in green. The colored blocks next to the tree indicate the Bayesian Analysis of genetic Population Structure (BAPS) clusters and sequence types (STs) that an isolate belongs to. The pie chart represents source niche of strains carrying each lineage-specific ST.

A total of 4498 *Salmonella* strains representing 89 serovars and harboring the complete MLST genes were collected to identify putative polyphyletic serovars (see Table S1). This data set was used to construct an unrooted maximum-likelihood (ML) tree of the MLST gene sequences. From this ML tree, the strains representing most serovars formed serovar-specific clades (Figure 1A). The strains of serovars Paratyphi B, Kentucky, Saintpaul, Newport, Muenchen, Montevideo, and Bareilly formed more than one separate cluster, suggesting that these serovars are putatively polyphyletic. There have been no comprehensive genome-wide analyses of the identified putative polyphyletic serovars Montevideo, Bareilly, Saintpaul, and Muenchen. The next steps were to characterize these four serovars based on WGS data.

### 2.2. Extensive Genetic Diversity Is Revealed by Whole-Genome-Based Phylogeny, Population Structure, and Average Nucleotide Identity (ANI)

To further investigate the relatedness among these four serovar strains and infer the evolutionary history of each strain, 347 *Salmonella* genomes were collected to construct the core genome phylogeny. The collection contained Montevideo (n = 60), Bareilly (n = 109), Saintpaul (n = 68), Muenchen (n = 37), other *S. enterica* subsp. *enterica* strains (n = 70), and three *S. diarizonae* strains as outgroup (Table S1). A maximum-likelihood (ML) tree was constructed based on the single-nucleotide variants for the 805 single-copy core genes (Table S2) shared by all 347 *Salmonella* strains. In the core genome tree, strains within each clade are highly clonal, as indicated by the short branch lengths (Figure 1B). Among four putative polyphyletic serovars, Montevideo and Muenchen form multiple distinct lineages (designated as multi-lineage serovar) (Figure 1B). Serovar Montevideo strains were classified into four highly clonal clades (designated MvPL-1 to 4) with different sequence types (STs) and two Bayesian Analysis of genetic Population Structure (BAPS) clusters, containing 6, 8, 3, and 43 strains in clades MvPL-1 to 4, respectively. Serovar Muenchen strains were clustered into two highly clonal clades (designated MhPL-1 and 2) and two BAPS clusters, each clade with 18 isolates. Furthermore, more than 700 unique SNPs for each of MvPL-1 to 4 and more than 1100 unique SNPs for MhPL-1 and 2 were also uncovered. These divergences were of a similar scale with that separated the serovars Enteritidis (host generalism) and Gallinarum/Pullorum (host restriction) in core genome tree. High phylogenetic diversity within Montevideo and Muenchen in combination with the above SNPs on each disparate clade indicate that multi-lineage serovars of *Salmonella* may be comprised of several genomically divergent and phylogenetically distinct clones.

The core genome tree reveals the polyphyletic nature of Bareilly and Saintpaul, and that all strains carrying serovar Bareilly or Saintpaul clearly do not share a recent common ancestor (Figure 1B). Most strains of Bareilly and Saintpaul were classified into two phylogenetic lineages (designated BPL-1 and 2, SPL-1 and 2) that were phylogenetically distinct from each other. BPL-1 contained fourteen isolates and five STs, grouped into one BAPS cluster. BPL-2 contained a majority of the Bareilly strains and various STs, with 94 isolates and seven STs, grouped in two BAPS clusters. SPL-1 contained 29 isolates and one ST, grouped into one BAPS cluster. SPL-2 contained 39 isolates with four STs, grouped into two BAPS clusters. Overall, genetically distinct lineages possess different STs.

The MLST tree based on the 347 strain set also exhibits multiple phylogenetic lineages of each of the four serovars (Figure S1). The ANI value was applied to estimate the genetic distance between strains at the whole-genome level [16]. The pairwise ANI values were calculated to examine the inter-lineage genetic relatedness within these four serovars. As shown in Figure S2, the inter-lineage ANI values are below the intra-lineage values, similar to the inter-serovar values, indicating the prominent genetic distance between distinct lineages within each of the four serovars. One Bareilly strain (*S.* Bareilly CFSAN000181: ST2555) and one Muenchen strain (*S.* Muenchen baa 1674: ST84) which are singletons (Figure 1B, highlighted in the grey block) were also collected. Due to the small amount of singleton data, only strains in the designated lineages will be analyzed in the following part.

### 2.3. Characterizing the Core and Pan Genomes Exhibits the Open Pan Genome and the Small Core Genome

**Figure 2:**
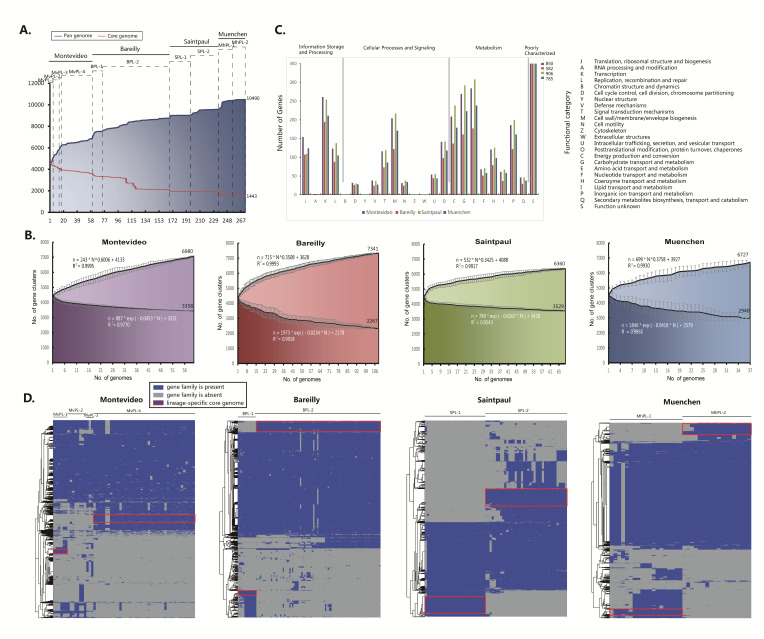
Core and pan genome analysis. (**A**) Core and pan genome curves for strains in lineages of Montevideo, Bareilly, Saintpaul, and Muenchen. (**B**) Increase and decrease in gene families in the pan genome and core genome. Black spots are the averages of each value. Error bars indicate standard deviation in the number of core and pan gene clusters among different strains. The deduced mathematical functions of core and pan genome curves are also reported. (**C**) Distribution of clusters of orthologous group (COG) catalogues of core gene families. (**D**) Cluster map of the accessory genome. The gene families that are unique to a lineage and conserved across most strains in that lineage are framed in red.

To assess the genetic diversity, the core and pan genome across Montevideo, Bareilly, Saintpaul, and Muenchen were characterized. Firstly, the core and pan genome curves for all strains of these four serovars were constructed (Figure 2A). A total of 10,490 gene families of pan genome were identified, 1443 of which were core gene families. The pan genome curve is noticeably shaped by the number of novel gene additions with each additional genome, especially if the additional genome belongs to a distinct lineage (Figure 2A). Similarly, a sudden drop in the core genome curve was observed for the novel additional genome from a distinct lineage (Figure 2A). This effect is due to the phylogenetic distance between distinct lineages. The pan and core genome analysis indicated that additional lineage-specific genomic content exist in these four serovars.

Furthermore, the pan and core genome curves for each of the four serovars were separately constructed (Figure 2B). In total, 3358 Montevideo, 2267 Bareilly, 3529 Saintpaul, and 2940 Muenchen core gene families were identified (Figure 2B). These core genes distributed unevenly across the functional categories (Figure 2C). As shown in Figure 2C, a larger proportion of the core genes of all four serovars were involved in the transcription (category K), transport and metabolism of carbohydrates and amino acids (categories G and E). A mathematical model was used to estimate the minimum number of core genes by fitting a single exponential decay function [17]. The core gene content of all four serovars decreased continuously as the number of genomes increased (Figure 2B), indicating that the core gene content reached a stable minimum, and additional strains would not substantially reduce the core gene content. The predicted minimum core gene content of Montevideo, Bareilly, Saintpaul, and Muenchen was 3358, 2267, 3529, and 2940 genes, respectively, which was reduced by 453, 1,544, 282 and 871 genes, respectively, than the core gene content of the monophyletic serovar Typhimurium (3811 genes) [15]. These results suggest that there are many gene content variations in serovars with multiple phylogenetic lineages.

The pan genomes of Montevideo, Bareilly, Saintpaul, and Muenchen contained 6980, 7341, 6360, and 6727 genes, respectively (Figure 2B). The pan genome content of these four serovars shows a clear linear upward trend consistent with Heap’s law pan genome model [18], and a robust fit of the data for all four serovars was obtained for the increasing power model with the positive exponent *γ* = 0.6006, 0.3508, 0.3425, and 0.3758 (Figure 2B). The exponent *γ* > 0 indicates an open pan genome [18]. The open pan genomes of these serovars revealed the genomic dynamics among each distinct lineage and the associated tendency to divergently evolve for adaptation to diverse niches. This observation is not surprising if a serovar possesses multiple phylogenetic lineages that are related to different source niches. The same result was observed for the polyphyletic serovar Paratyphi B, in which the pan genome is open and divergent evolution between distinct lineages is associated with a diverse disease phenotype [11].

### 2.4. The Source Niche Metadata and Lineage-Specific Genomic Contents Reveal the Potential Differentiation in Niche Adaptation

Previous studies revealed that certain *Salmonella* lineages have preferred niche ranges, and explored this aspect of *Salmonella* biology by source attribution [10,19]. To investigate the possible lineage-specific niche preference in a broad context, the EnteroBase database was acquired for the source niche metadata for 6142 strains carrying the given STs of Montevideo, Bareilly, Saintpaul, and Muenchen (see Table S3). Interestingly, it is found that the distribution of host and environmental isolates is distinct in the genealogy. Montevideo and Muenchen strains carrying STs of MvPL-2 and MhPL-2 appear to be predominantly associated with poultry (76.7% and 86.3%, respectively), those carrying STs of MvPL-1 appear to be predominantly associated with human and poultry (34.5% and 32.1%, respectively), and those carrying STs of MvPL-3 and MhPL-1 appear to predominantly associated with the environment (75.1% and 63.3%, respectively). MvPL-4 contains isolates from environment, food, poultry, and human (38.3%, 13.9%, 23.4%, and 21.3%, respectively). Similarly, for two polyphyletic serovars, namely, Bareilly and Saintpaul, strains carrying STs of BPL-1 and SPL-1 appear to be predominantly associated with environment (78.9% and 85.4%, respectively), however, those carrying STs of BPL-2 appear to be predominantly associated with human (78.9%), and those carrying STs of SPL-2 appear to be predominantly associated with human and poultry (40.0% and 44.8%, respectively). Taken together, these different proportions of source niche metadata in distinct lineages indicate potential lineage-specific niche preference. MvPL-1, MvPL-2, BPL-2, SPL-2, and MhPL-2 are associated with host source and can be described as potential host-preferred lineages. The other lineages, namely, MvPL-3, MvPL-4, BPL-1, SPL-1, and MhPL-1 are associated with environmental sources and can be described as environment-preferred lineages. The differences in niche preference may indicate the divergent evolution of niche adaptation and epidemiology at the intra-serovar level of Montevideo, Bareilly, Saintpaul, and Muenchen. It is worth noting that the potential sampling bias in the EnteroBase database might lead to distortion of source niche. Future studies are required to further confirm these potential lineage-specific niche preferences and their correlation with biological characteristics.

**Figure 3:**
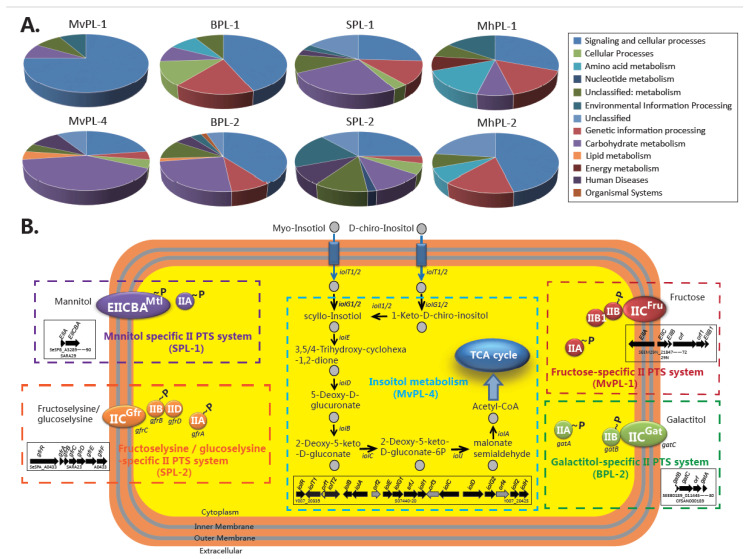
Functional enrichment of the lineage-specific genomic contents in the KEGG annotation. (**A**) Detailed enrichment results of the lineage-specific genomic contents based in the KEGG annotation. (**B**) Complete pathway modules in the lineage-specific genomic contents.

Niche preferences may be associated with lineage-specific genomic content. The cluster heatmaps (Figure 2D) of the accessory genomes of four selected serovars reveal that each distinct lineage is differentiated by a set of lineage-specific conserved gene families (framed in red, Figure 2D). MvPL-1 to 4, BPL-1 and 2, SPL-1 and 2, and MhPL-1 and 2 had 43, 1, 4, 66, 55, 162, 177, 135, 77, and 133 lineage-specific gene families (see Table S4), respectively, which were assigned according to the KEGG database. The KEGG functional classification is shown in Figure 3A, in which “metabolism”, “cellular processes”, and “genetic information processing” are the major functional categories. The functional categories involved in metabolism were enriched in the lineage-specific genomic contents, which indicated the differences in metabolic abilities and niche adaptation between distinct lineages.

There are several complete pathway modules in the lineage-specific genomic contents of these four serovars, which are related to carbohydrate transport and metabolism (Figure 3B). For the host-preferred lineages, a putative fructose phosphotransferase system (PTS) encoding locus is present in MvPL-1, but is absent in other lineages, and may be involved in fructose utilization [20]. Four components (EIIA, EIIB, EIIB1, and EIIC) constitute this putative fructose PTS. Fructose is a major component of many diets suggesting that fructose utilization could contribute to the fitness of MvPL-1. The BPL-2 strains contain a galactitol-specific PTS comprised of EIIA, EIIB, and EIIC, which enable galactitol uptake [21]. Recent studies have indicated that utilization of galactitol contributes to the proliferation of *S. enterica* strains [21,22]. In this study, the utilization of galactitol as a BPL-2-specific metabolic profile is considered critical to host adaptation. In addition, SPL-2 possessed fructoselysine/glucoselysine PTS, indicating that these strains could utilize fructoselysine and glucoselysine as carbon and nitrogen sources. Fructoselysine is common in dehydrated fruits, grains, and vegetables. Enteric bacteria may encounter fructoselysine from glycated proteins in the host diets [23]. Ali et al. recently showed that utilization fructose-asparagine was essential for *Salmonella* fitness in an inflamed intestinal model [24]. It is speculated that fructoselysine may also be a nutrient and confer a fitness advantage for SPL-2 in host intestine.

In the environment-preferred lineages, the PTS for mannitol uptake and phosphorylation, comprising EIICBA and EIIA, is present only in the SPL-1 strains (Figure 3B). Mannitol is a polyol produced by marine algae and is the most abundant hexitol found in fungi, algae, and plants [25,26]. Mannitol metabolism has been intensively studied in marine bacteria, such as *Vibrio cholerae* and *Zobellia galactanivorans* [26,27]. Furthermore, mannitol is widely used in food, pharmaceutical, medical and chemical industries [28]. MvPL-4 has a full pathway for utilization of *myo*-inositol (MI), a polyol that can serve as the sole carbon and energy source of *S.* Typhimurium (Figure 3B) [29,30]. MI is ubiquitously present in environments harboring salmonellae, such as soli and plants, where it spears as a free form or as phospholipid derivatives [30]. It is worth noting that the Inositol utilization island is absent in the genomes of host-restricted serovars like Typhi, Paratyphi A, and Choleraesuis [31]. These results indicate that the mannitol transport system in SPL-1 and utilization of inositol in MvPL-4 may have an effect on the preferred environment niche of the lineage.

### 2.5. The Virulence Profile Indicates the Divergence in Pathogenicity between Distinct Lineages

**Figure 4:**
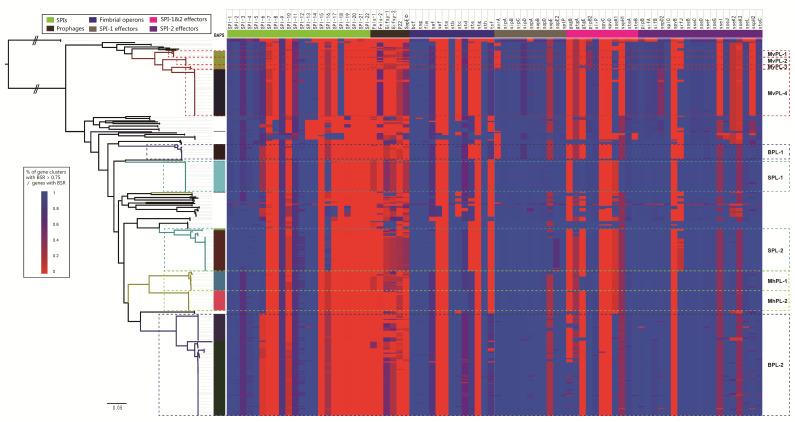
Heatmap of the distribution of SPIs, prophage, fimbrial operons, and effectors. Color coding for the gene clusters (SPIs, prophage, and fimbrial operons) is based on the percentage of genes on a cluster that are present in a genome (defined as the Blast score ratio (BSR) of query gene > 0.75). Color coding for effectors is based on the Blast score ratios calculated when the genomic data were screened against the effectors.

Many of the virulence phenotypes of *S. enterica* are encoded by virulence-related genetic elements. To investigate the pathogenic variation at the intra-serovar level, the key genomic characteristics, including SPIs, prophages, fimbrial operons, and type three secretion system (T3SS) effectors, were analyzed. Some genetic variations between distinct lineages within the same serovars were observed. Regarding SPIs, SPI-6 comprised of the type six secretion system (T6SS), the saf fimbrial gene cluster and the invasin *pagN*, which are all present in SPL-2; however, the T6SS of SPI-6 is missing in SPL-1 (Figure 4). Pezoa et al. demonstrated that the T6SS in SPI-6 was crucial for gastrointestinal colonization of chicks by *S.* Typhimurium [32]. Notably, approximately 45% of the strains of SPL-2 (host-preferred lineage) were isolated from poultry. The presence of T6SS suggests that this system may contribute to the preferred poultry niche of SPL-2.

Variations in the fimbrial content of Bareilly and Saintpaul were also observed at the inter-lineage level. The ste operon is found in BPL-2 and SPL-1, but is absent in BPL-1 and SPL-2 (Figure 4). Furthermore, there are some variations in T3SS effectors (Figure 4). BPL-2 possesses the SPI-1 effector *avrA* and SPI-2 effector *srfJ*, whereas isolates of BPL-1 lack these effectors. Similarly, it is observed that *gtgE* and *srfJ* are present in all isolates of SPI-1. It is also found that all isolates of MvPL-4 possess the effector *avrA*, whereas members of other lineages of Montevideo have lost this effector. Although these genomic variations in fimbrial content and effectors are not associated with ecological niche, these variations suggest a difference in pathogenicity at the inter-lineage level in a serovar. Future studies are required to evaluate the function of these variations and their correlation with pathogenicity and epidemiology. Furthermore, these genomic variations could be used as the monitoring targets for identification these host lineages using PCR.

### 2.6. Differences in the Antimicrobial Resistance (AMR) Profile at the Inter-Lineage Level within Saintpaul and Muenchen

**Figure 5:**
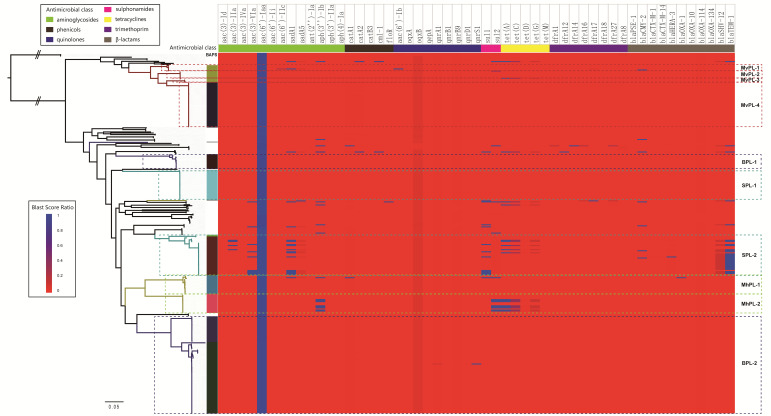
Heatmap of the distribution of antimicrobial resistance genes.

Using WGS data, it is possible to compare the resistance elements (Table S5) of the serovars Montevideo, Bareilly, Saintpaul, and Muenchen at the intra-serovar level. There is a lack of resistance genes in the Montevideo, Bareilly, Saintpaul, and Muenchen (Figure 5). Resistance genes were acquired via single, local events that occurred in SPL-2 and MhPL-2. Based on the whole-genome prediction, genotypic resistance to aminoglycosides, sulphonamides, tetracyclines, and β-lactams was detected in 13 (33.3%), 8 (20.5%), 5 (12.8%), and 26 (66.7%) isolates of SPL-2, respectively (Figure 5). Most of these isolates encoded genes to at least one antimicrobial class. In addition, 8 of the 18 isolates in MhPL-2 were multidrug resistant (MDR), representing the consistent genotypic resistance [aph(3′’)-Ib/sul2/tet(A)] to aminoglycosides, sulphonamides, and tetracyclines. Overall, SPL-2 and MhPL-2 as host-preferred lineages, possess extensive AMR profiles, indicating that the AMR profiles are also part of the lineage-specific genomic content and associated with niche preference.

### 2.7. Polyphyletic Serovars Are the Result of Recombination Events at the H1-Antigen Loci

**Figure 6:**
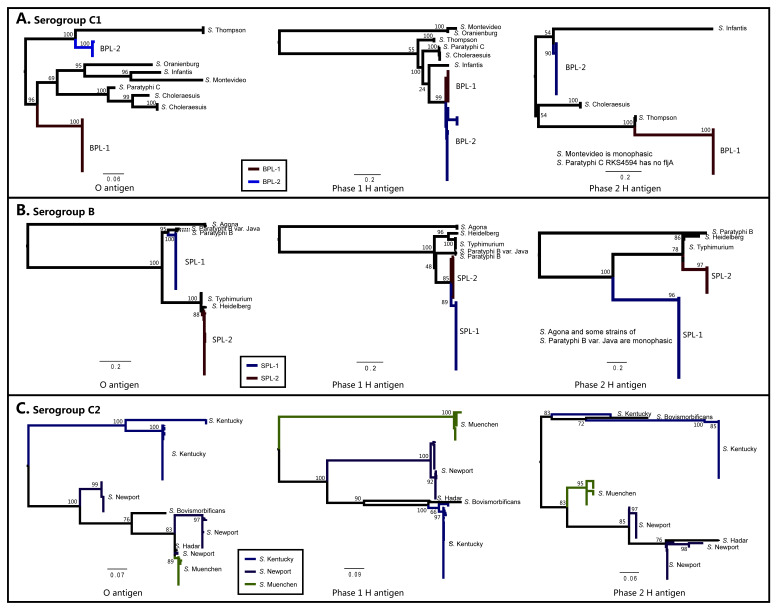
Phylogenetic analysis of O-, H1- and H2-antigens. Maximum-likelihood trees based on the nucleotide sequences of core genes in O-, H1- and H2-antigen clusters of the strains belong to serogroup C1 (**A**), B (**B**), and C2 (**C**), drawn using MEGA 7 software with GTR model. The primary node values of the tree are the bootstrap values (100 replicates). The genes used for the analysis were listed in Table S6.

The cause of the common serovars between distinct phylogenetic lineages need to be explored. Connor et al. suggested that the genetic variability in the polyphyletic serovar, Paratyphi B, may be due to recombination at the flagellum loci [11]. Thus, the genetic variability of Bareilly (serogroup C1), Saintpaul (serogroup B), and two previous studied polyphyletic serovars by WGS analysis (Newport (serogroup C2) and Kentucky (serogroup C2)) [12,13] were further investigated. Both the O-antigen and H2-antigen trees of each serovar reveal a similar topology to the core genome trees (Figure 1B; Figure 6; Figure S3) and form multiple phylogenetic lineages. Similar to the core genome tree, the distant phylogenetic lineages of O and H2-antigen trees of polyphyletic serovars exhibit a low level of relatedness in genetic aspects, implying a distinct evolutionary history. In contrast, the phylogenetic topology of the H1-antigen of polyphyletic serovars (Figure 6) individually reveals marked differences from those of the core genome and O- and H2-antigen gene clusters. In the case of the H1-antigen trees, the strains are gathered to a monophyletic group and originated from a common ancestor. These polyphyletic serovars are manifested in the lack of diversity within the H1-antigen (Figure 6; Figure S3B); the likely explanation is the occurrence of recombination. Homologous recombination occurs in bacterial populations and can lead to switching of genetic backgrounds. These results indicate that recombination event at H1-antigen loci is a source of polyphyletic serovars.

### 2.8. Polyphyletic and Multi-Lineage Serovars Are Clinically Important Salmonella Serovars

The U. S. Centers of CDC supports foodborne disease surveillance and provides the *Salmonella* annual report. A total of 46,623 cases of culture-confirmed *Salmonella* infections were reported to the Laboratory-based Enteric Disease Surveillance (LEDS) system, United States, 2016. Table 1 listed the 20 most frequently reported serovars. Montevideo, Bareilly, Saintpaul, and Muenchen caused 1018, 412, 778, and 1216 illnesses in the USA (total = 32,271) in 2016, respectively (Table 1). As one of the top 10 most common serovars, Montevideo is associated with contaminated foods, including black and red peppercorn, tahini, and pistachio [19,33,34], and is linked to more than 240 illnesses in 38 states with contaminated Italian-style spiced meats [34,35]. Bareilly, first identified in India in 1928, is known for its wide host range and has been associated with multiple multistate outbreaks, for instance, a widespread foodborne outbreak in the United States associated with scraped tuna imported from India [36]. The serovar Saintpaul was first isolated in the United States in 1940 [37] and considered to be a major source of salmonellosis worldwide, being responsible for recent outbreaks in the United Kingdom [38], Denmark [39], Australia [40], Germany [41] and the United States. Muenchen also ranks among the top 10 most common serovars and is associated with multiple multistate outbreaks [42,43]. The occurrence of MDR Muenchen strains has been reported [44]. Furthermore, two reported polyphyletic serovars, namely, Newport and Paratyphi B, are ranked second and 19th among the top 20 serovars (Table 1). These serovars with multiple lineages of clinically important Salmonella serovars present public health concerns. Whole-genome-based accurate subtyping methods provide the resolution needed for epidemiological investigations.

### 2.9. CRISPR-Cas System: A High-Resolution Subtyping Method for Polyphyletic and/or Multi-Lineage Serovar

**Figure 7:**
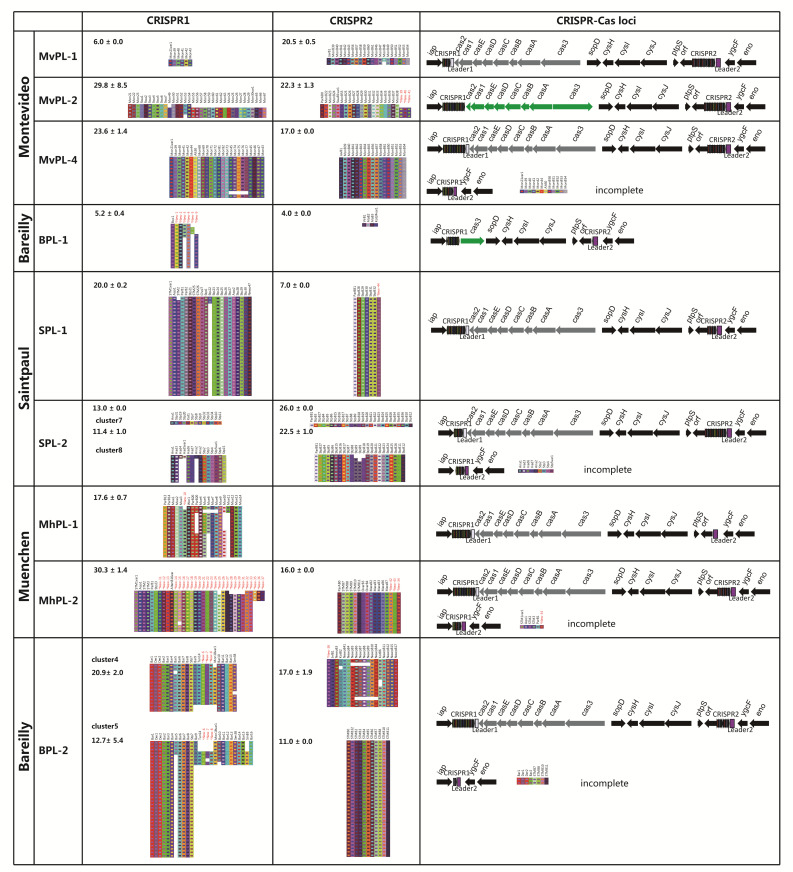
Structures of CRISPR-Cas systems from Montevideo, Bareilly, Saintpaul, and Muenchen. The organizations of the CRISPR-Cas system in each lineage is arranged in the order of the core genome tree. The *cas* genes of the LT2 subtype are represented by the grey arrows, and the ty2 subtype is represented by the green arrows. Leader1 is represented by the white square, and the purple square represents Leader2. Colored squares under the gene clusters represent spacer organization. Spacers of the same color and symbols indicate sequence consistency. The values shown are the mean (±SD) number of spacers per array. The known and new spacers are marked with the spacer ID in Institut Pasteur and asterisk, respectively. The new spacers also are indicated in red. The different BAPS clusters in an individual lineage are labelled. Samples are in the same order as they are in the core genome tree (Figure 1B).

CRISPR-Cas system might provide effective information that is useful for typing [46,47,48]. To evaluate the microevolution of the CRISPR-Cas system at the intra-serovar level, sequence analysis of the CRISPR-Cas systems across these four serovars were performed. By comparison of these spacers and the known spacers implemented in Institut Pasteur [48], 304 different spacers were found, including 260 known spacers and 44 new spacers (Figure 7). There are 192 and 122 different spacers in CRISPR1 and CRISPR2, respectively, among these four serovars. All the CRISPR1 and CRISPR2 spacer arrays identified are shown in Figure 7. Strains within an individual lineage have very similar spacer content and identical orders within the arrays for both CRISPR1 and CRISPR2. Each phylogenetic lineage exhibits a distinct spacer content and organization of both CRISPR1 and CRISPR2. Only a few shared spacers were found among distinct lineages in an individual serovar. In CRISPR1, MvPL-1, BPL-2, SPL-1, and MhPL-2 possess a greater number of spacers than other lineages of each serovar. In CRISPR2, SPL-2 possess more spacers than SPL-1. Specifically, MhPL-1 and most strains of BPL-1 do not contain CRISPR2. Furthermore, there are two distinct sets of CRISPR arrays in both SPL-2 and BPL-2 (Figure 7), which are subdivided into two clusters by BAPS in the core genome tree.

Most strains within an individual lineage have identical *cas* gene clusters (Figure 7). Most lineages possess the complete *cas* gene cluster of the LT2 subtype. However, the type and content of *cas* gene clusters present in each lineage of Montevideo and Bareilly exhibit some differences. MvPL-2 has complete *cas* gene clusters of the ty2 subtype, while BPL-1 loses most *cas* genes and retains only a portion of *cas3* of the ty2 subtype. Moreover, Leader1 and Leader2 were detected in most strains across all four serovars, however, Leader1 was not detected in MvPL-2. There is a detected deletion of a large segment downstream from downstream from CRISPR1 to upstream from Leader2. Such deletion was also observed in a few strains of MvPL-1, SPL-2, MhPL-2, and BPL-2 (Figure 7, mentioned “incomplete”). To summarize, the divergence of the three functional elements (CRISPR array, *cas* gene cluster, and leader sequence) that comprise the CRISPR-Cas system between distinct lineages across Montevideo, Bareilly, Saintpaul, and Muenchen were analyzed. These results demonstrate that lineage-specific features of the CRISPR-Cas system can be used for discrimination among distinct lineages carrying the common serovar.

## 3. Conclusions

This work focused on four special serovars listed in the CDC’s top 20 serovars. According to the whole-genome analysis, the results provide a complete view of the genetic diversity and evolutionary relationships of the multi-lineage Montevideo and Muenchen, and the polyphyletic Bareilly and Saintpaul, which will provide the accurate subtyping for future taxonomic and functional genomics studies of these strains. Notably, all four serovars and two previously studied polyphyletic serovars, namely, Newport and Paratyphi B, are consistently ranked in the CDC’s list of the top 20 *Salmonella* serovars attributed to human illness in the USA. To avoiding misleading interpretations regarding the evolutionary relatedness of strains due to serotyping, this work represents an insight into pathogenicity investigation and epidemiological surveillance of *S. enterica* subspecies *enterica*. This work also characterized the lineage-specific genetic contents, which reveals the divergence towards niche adaptation, pathogenicity, and antimicrobial resistance occurred in distinct lineages. These results reveal that some polyphyletic serovars are the result of recombination events at H1-antigen loci. WGS provides detailed genomic information for epidemiological tracking and will yield invaluable insights into the accurate evolutionary relatedness of bacterial pathogens, especially for *S*. *enterica* subsp. *enterica*.

## 4. Materials and Methods

### 4.1. Data Collection

All the *S. enterica* genomes (n = 5391) were downloaded from the NCBI GenBank database in March 2017. First, the non-*enterica* subspecies strains were excluded. Second, only strains with annotations and serovar naming were selected for further filtering. Third, strains missing at least one of the seven housekeeping genes (*aroC, dnaN, hemD, hisD, purE, sucA*, and *thrA*) used in the MLST scheme were excluded. Fourth, to avoid potential mistakes resulting from serotyping errors, a verification process for serotyping was created. To obtain the STs (sequence types), the nucleotide sequences of the MLST genes were aligned against sequence data from the EnteroBase database. The composite STs were defined by the EnteroBase database (http://mlst.warwick.ac.uk/mlst/dbs/Senterica/) based on the set of allelic profiles derived from each of the seven loci. STs were often correlated with serovars [5]. For the selected strains, the relationships between the STs and serovars should be consistent with their NCBI annotations (serovar annotations in file: assembly summary.txt). Finally, a total of 4498 *S. enterica* subsp. *enterica* strains were selected for comparative genomic analysis (see Table S1). The serotyping of these strains was further confirmed by using SeqSero2 [49]. The excluded strains are listed in Table S7. EnteroBase was also queried for the source niche of the strains (n = 6142) of the given STs of Montevideo, Bareilly, Saintpaul, and Muenchen [50] (listed in Table S3).

### 4.2. Construction of MLST Tree

Nucleotide sequences of the 7 MLST genes were aligned using MAFFT [51] with default parameters. Phylogenetic trees were constructed by Maximum likelihood method with MEGA 7 software [52], (with the General Time Reversible (GTR) model). FigTree 1.4.3 were employed to show the trees.

### 4.3. Identification of Gene Orthologous Group

Orthologous groups were delimited using OrthoFinder [53], in which all the protein sequences were compared using a BLASTP all-against-all search with an E-value cutoff of <10^−3^. The single–copy core gene, pan genome and core genome sets were extracted from the OrthoFinder output. Nucleotide sequences of the single-copy core genes were extracted according to protein ID.

### 4.4. The Phylogenetic Analysis and Population Genetic Analysis Based on Core Genome Single-Nucleotide Variants (cgSNVs)

A total of 347 *Salmonella* strains, including 68 Saintpaul, 109 Bareilly, 37 Muenchen, 60 Montevideo genome sequences, 70 other serovars genome sequences, and three outgroup *Salmonella enterica* subsp. *diarizonae* genome sequences were used in this phylogenetic analysis. The nucleotide sequence of genes using in phylogenetic analysis were aligned using MAFFT [51]. The phylogenetic analyses were constructed by the set of cgSNV present in all single-copy core genes across genomes. The SNPs were integrated according to the arrangement of the single-copy genes on LT2 reference genome. In consideration that homologous recombination caused by horizontal gene transfer occurring in bacterial populations and can confound phylogenetic analysis. Putative recombined regions of the set of cgSNV were identified and removed, using CloneFrameML [54].The ML tree was constructed using MEGA 7 software [52] (with the GTR model and 100 bootstrap).

The software package BAPS [54] was used to analyze population structure, based on SNPs identified from the alignment of the single-copy core genes. BAPS assigns strains to inferred population (K) representing the best fit for the observed genetic variation. K was varied from 5 to 40 and ran three times to confirm the clustering results.

### 4.5. Whole Genome Average Nucleotide Identity

The ANI value was calculated for 347 strain set using JSpecies 1.2.1 [16], using the ANIm method with default parameters. The result was visualized using the pheatmap R packages (see Figure S2).

### 4.6. Core and Pan Genome Analysis

The regression analysis for the core gene cluster curve was performed a weighted least square regression by fitting the power law *n* = *к*exp(*m* ×N) + *Θ* to means [55]. *N* is the number of genomes, *n* is the number of core gene clusters, *Θ* is a constant value representing the predicted minimum number of core genes, and *к* and *m* are parameters. According to the Heap’s law pan genome model described in reference [18], the total number of gene clusters is shown for increasing values of the number *N* of genomes. The curve was a least squares fit of the power law *n* = $кN^{\gamma }$ to averages. The exponent *γ* > 0 indicates an open pan genome serovar. The functional category of core gene clusters was performed by alignment against the cluster of orthologous groups (COG) database of NCBI using BLASTp with an E-value of 10^−6^.

### 4.7. Lineage-Specific Core Genome Comparison

To exhibit the pan genome more intuitive, a cluster heatmap for the gene families all four serovars was constructed using the heatmap clustering command from the pheatmap R packages (Figure 3). The core gene families and low frequency gene families that are shared by less than 10 strain genomes were excluded. The results were designed as “the lineage-specific core genome” (see Table S4), which represents the set of gene families that are shared across all strains of a lineage and absent in other lineages. This approach was described previously [56]. The functional analysis of lineage-specific core genome was done according to the KEGG database.

### 4.8. Identification of Virulence-Related Elements and Antimicrobial Resistance Genes

To examine SPI-1 to -22, prophage, fimbrial operon, and effectors, gene or gene cluster were located and screened using the LS-BSR tool with default parameters [57]. The reference database included acquired resistance genes and mutations conferring resistance to seven antimicrobial classes (aminoglycosides, phenicols, quinolones, sulphonamides, tetracyclines, trimethoprim, and β-lactams) [11,58]. The nucleotide sequences of reference resistance genes (see Table S5) were downloaded from the Resfinder 2.0 database [59] and the ARDB 1.1 Database [60]. To identify the resistance genes, the genomes were aligned using BLASTn with an E-value cutoff < 10^−6^, identity > 60%, and coverage > 60% against the dataset of reference resistance genes.

### 4.9. Phylogenetic Analysis of O, H1, and H2-Antigen

To construct the phylogeny, the core genes across the O, H1, and H2-antigen gene clusters were extracted, respectively. The core gene name across the O, H1, and H2 antigen gene clusters of serogroup C1, B, and C2 was listed in Table S6. The genes coding for O antigen synthesis are normally present as a gene cluster in the genome, which maps between *galF* and *gnd* in *Salmonella* [61]. The details of the genetic structure of *Salmonella* O-antigens were reviewed previously [61]. The extracted sequences were aligned with MAFFT [51], and were generated trees using MEGA 7 [52], (with the GTR model).

### 4.10. CRISPR-Cas Analysis

The complete CRISPR-Cas system which was located between two conserved genes (*iap* and *eno*), was identified. Strains which were unable to extract complete sequences of CRISPR-Cas system were removed from the analysis (including all three strains of MvPL-3). Putative DRs and spacers matches identified using CRISPR Recognition Tool (CRT1.2) [62] with default parameters. The identified spacers were compared to the known spacers scheme implemented in Institut Pasteur [48]. Two leaders in *S. typhimurium* LT2 genome were located, and then the program BLASTn was used to subsequently identify the leaders in the collection.

## Figures and Tables

**Table 1:** List of top 20 *Salmonella* serovars attributed to human illness in the USA^a^.

Rank.	Serovar	Serogroup	Number Reported (Total = 32,271)	per 100,000	MLST Phylogeny	Core Genome Phylogeny	MSTree of MLST Database	Recombination Events
**1**	Enteritidis	D	7830	16.8	Monophyly *	-	Monophyly	-
**2**	Newport	C2	4728	10.1	Polyphyly	Polyphyly [9]	Polyphyly	Recombination of H1 (This study)
**3**	Typhimurium	B	4581	9.8	Monophyly	-	Monophyly	-
**4**	Javiana	D	2719	5.8	Monophyly	-		-
**5**	I 4, [5],12:i:-	B	2179	4.7	Monophyly	-	Monophyly	-
**6**	Infantis	C1	1281	2.7	Monophyly *	-	Monophyly	-
**7**	Muenchen	C2	1216	2.6	Polyphyly	Multi-lineage (This study) Polyphyly [6]	Monophyly	-
**8**	Montevideo	C1	1018	2.2	Polyphyly	Multi-lineage (This study) Polyphyly [45]	Polyphyly	-
**9**	Braenderup	C1	1001	2.1	Monophyly	-	Monophyly	-
**10**	Thompson	C1	792	1.7	Monophyly	-	-	-
**11**	Saintpaul	B	778	1.7	Polyphyly	Polyphyly (This study)	Polyphyly	Recombination of H1 (This study)
**12**	Heidelberg	B	754	1.6	Monophyly	-	Monophyly	-
**13**	Oranienburg	C1	692	1.5	Monophyly *	-	Polyphyly	-
**14**	Mississippi	G	536	1.1	-		-	-
**15**	Typhi	D	423	0.9	Monophyly	-	Monophyly	-
**16**	Bareilly	C1	412	0.9	Polyphyly	Polyphyly (This study) Polyphyly [45]	-	Recombination of H1 (This study)
**17**	Berta	D	369	0.8	Monophyly	-	-	-
**18**	Agona	B	362	0.8	Monophyly	-	Monophyly	-
**19**	Paratyphi B var. L(+) tartrate+	B	343	0.7	Polyphyly	Polyphyly [11]	Polyphyly	Recombination of H [11]
**20**	Anatum	E1	257	0.6	Monophyly	-	-	-

^a^ National Enteric Disease Surveillance: *Salmonella* Annual Report, 2016. https://www.cdc.gov/nationalsurveillance/pdfs/2016-Salmonella-report-508.pdf. (Page 5). ^*^ These monophyletic serovar strains formed one cluster in our MLST tree, however, the singleton strain was also collected (Figure 1A).

## Supplementary Materials

The following are available online at https://www.mdpi.com/1422-0067/21/15/5226/s1, Figure S1: MLST tree of 347 *Salmonella* genomes, Figure S2: Heatmap of average nucleotide identity based on whole genome alignments of 347 *Salmonella* genomes, Figure S3: A: Core genome tree of serogroup C2 strains (n = 232). B: ML tree of H1-antigen gene cluster (*fliA*, *fliC*, *fliD*, and *fliS*) of 284 strains, Table S1: Genetic characteristics of strains in the current study and Serovar information included in each analysis, Table S2: List of 805 single-copy genes shared by 347 *Salmonella* strains, Table S3: Source niche information and distribution for Montevideo, Bareilly, Saintpaul, and Muenchen from the EnteroBase database, Table S4: List of lineage-specific genomic contents with KEGG annotation, Table S5: List of resistance genes screened across the *Salmonella* genomes in this study, Table S6: List of the core gene across the O-, H1-, and H2-antigen gene clusters of serogroups C1, B, and C2, Table S7: List of the excluded strains.

## Abbreviations

WGS	whole-genome sequencing
MLST	multi-locus sequence typing
CDC	Centers for Disease Control and Prevention
NCBI	National Center for Biotechnology Information
SPIs	*Salmonella* pathogenicity islands
ML	maximum-likelihood
ANI	average nucleotide identity
STs	sequence types
T3SS	type three secretion system
T6SS	type six secretion system
AMR	antimicrobial resistance
MDR	multidrug resistant
GTR	General Time Reversible
cgSNVs	core genome single-nucleotide variants
