# Supplementary material for: Whole-Genome-Based Survey for Polyphyletic Serovars of Salmonella enterica subsp. enterica Provides New Insights into Public Health Surveillance
Source: Int J Mol Sci. 2020 Jul 23;21(15):5226. doi: 10.3390/ijms21155226 (PMC7432358; doi:10.3390/ijms21155226)
Supplement: Supplementary file 1 [file ijms-21-05226-s001.zip › Supplementary Files/Supplementary legends.docx]

**Supplementary Figure legends**

Figure S1. MLST tree of 347 *Salmonella* genomes.

Figure S2. Heatmap of average nucleotide identity based on whole genome alignments of 347 *Salmonella* genomes.

Figure S3. A: Core genome tree of serogroup C2 strains (n = 232). B: ML tree of H1-antigen gene cluster (*fliA*, *fliC*, *fliD*, and *fliS*) of 284 strains.

Table S1. Genetic characteristics of strains in the current study and Serovar information included in each analysis.

Table S2. List of 805 single-copy genes shared by 347 *Salmonella* strains.

Table S3. Source niche information and distribution for Montevideo, Bareilly, Saintpaul, and Muenchen from the EnteroBase database.

Table S4. List of lineage-specific genomic contents with KEGG annotation.

Table S5. List of resistance genes screened across the *Salmonella* genomes in this study.

Table S6. List of the core gene across the O-, H1-, and H2-antigen gene clusters of serogroups C1, B, and C2.

Table S7. List of the excluded strains.
