# Supplementary figures and images for: Whole-Genome-Based Survey for Polyphyletic Serovars of Salmonella enterica subsp. enterica Provides New Insights into Public Health Surveillance
Source: Int J Mol Sci. 2020 Jul 23;21(15):5226. doi: 10.3390/ijms21155226 (PMC7432358; doi:10.3390/ijms21155226)

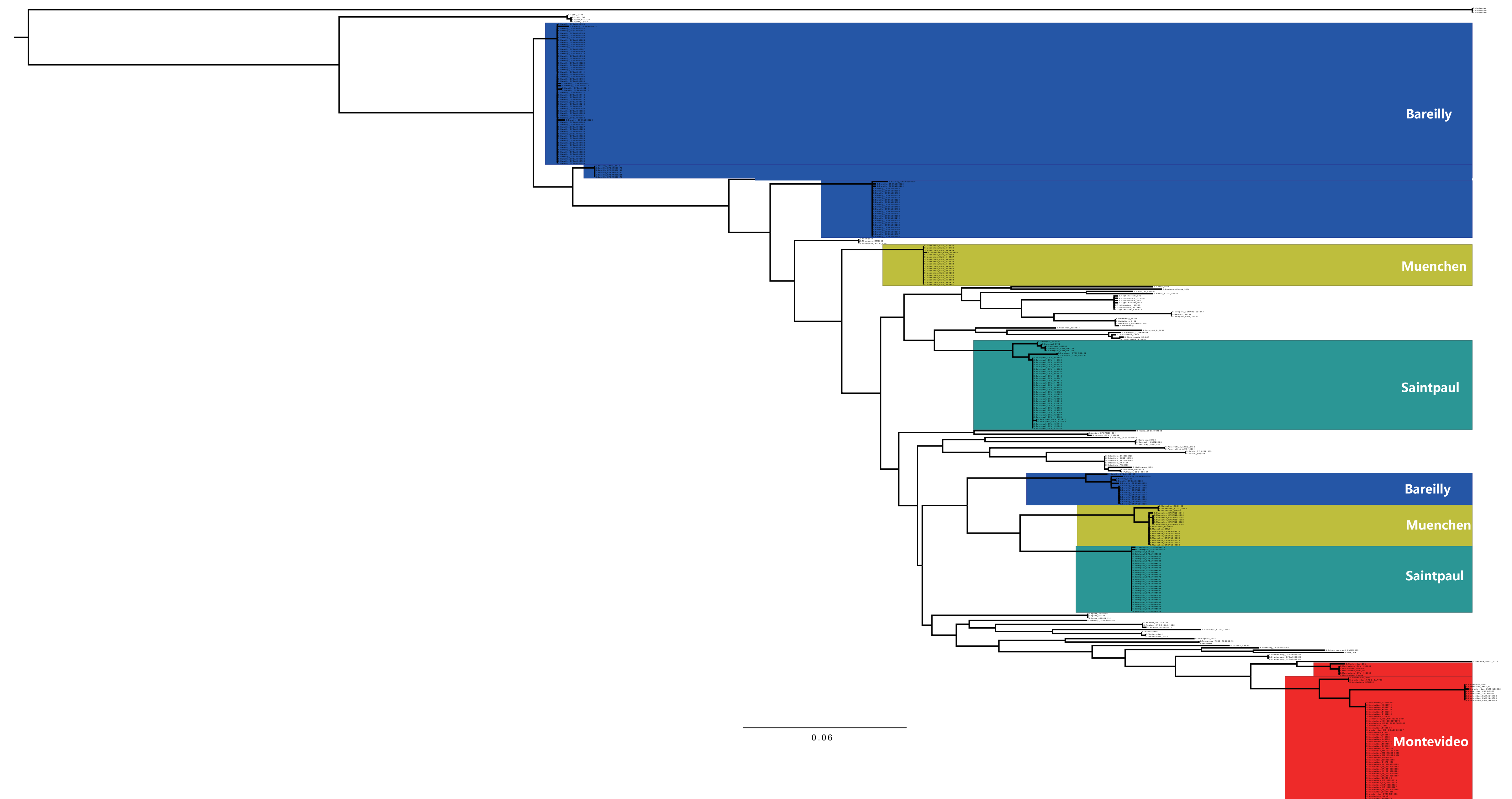

Supplement: Supplementary file 1 [file ijms-21-05226-s001.zip › Supplementary Files/Supplementary Figure S1.pdf]

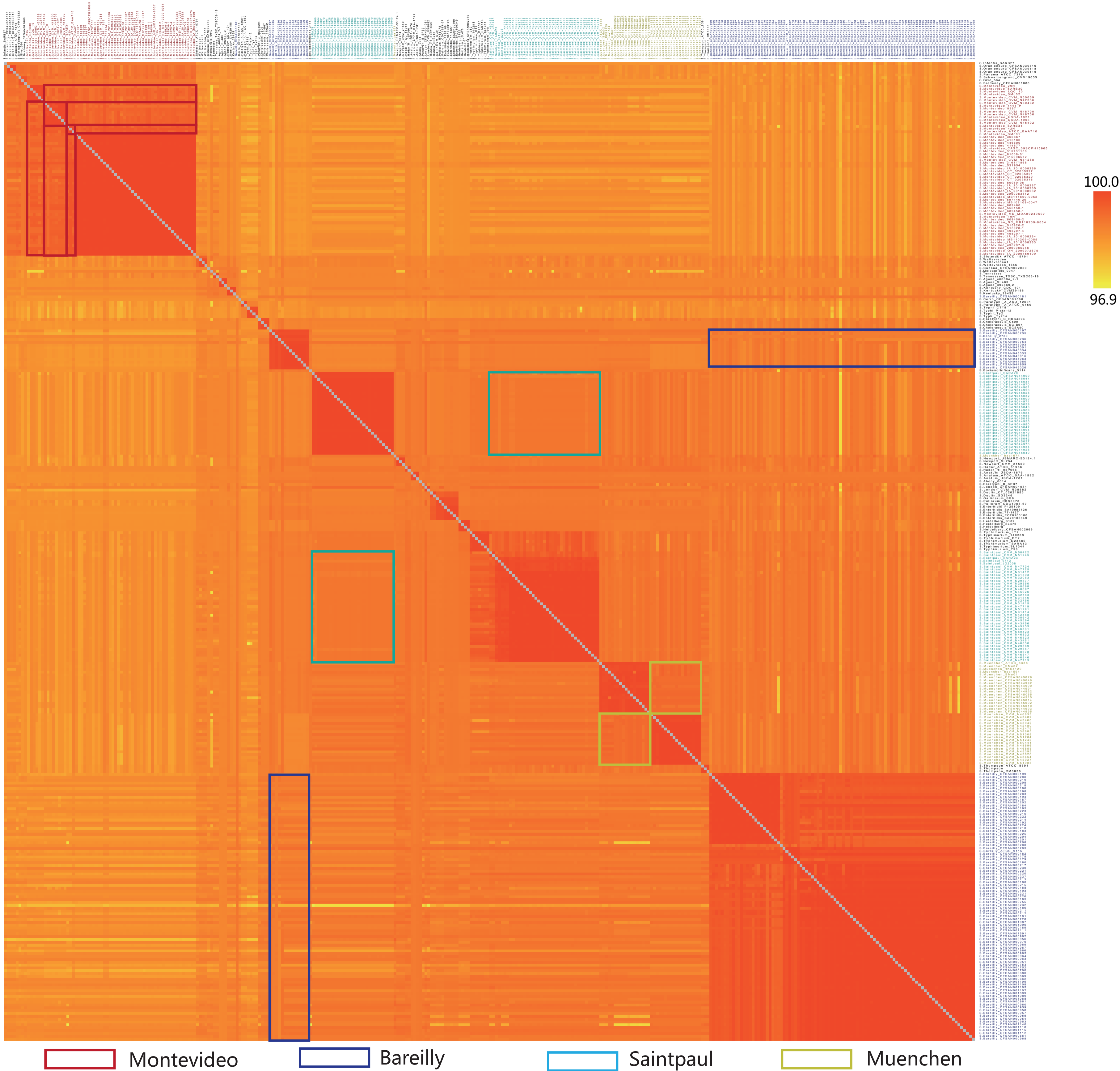

Supplement: Supplementary file 1 [file ijms-21-05226-s001.zip › Supplementary Files/Supplementary Figure S2.pdf]

## A Core genome tree of Serogroup C2

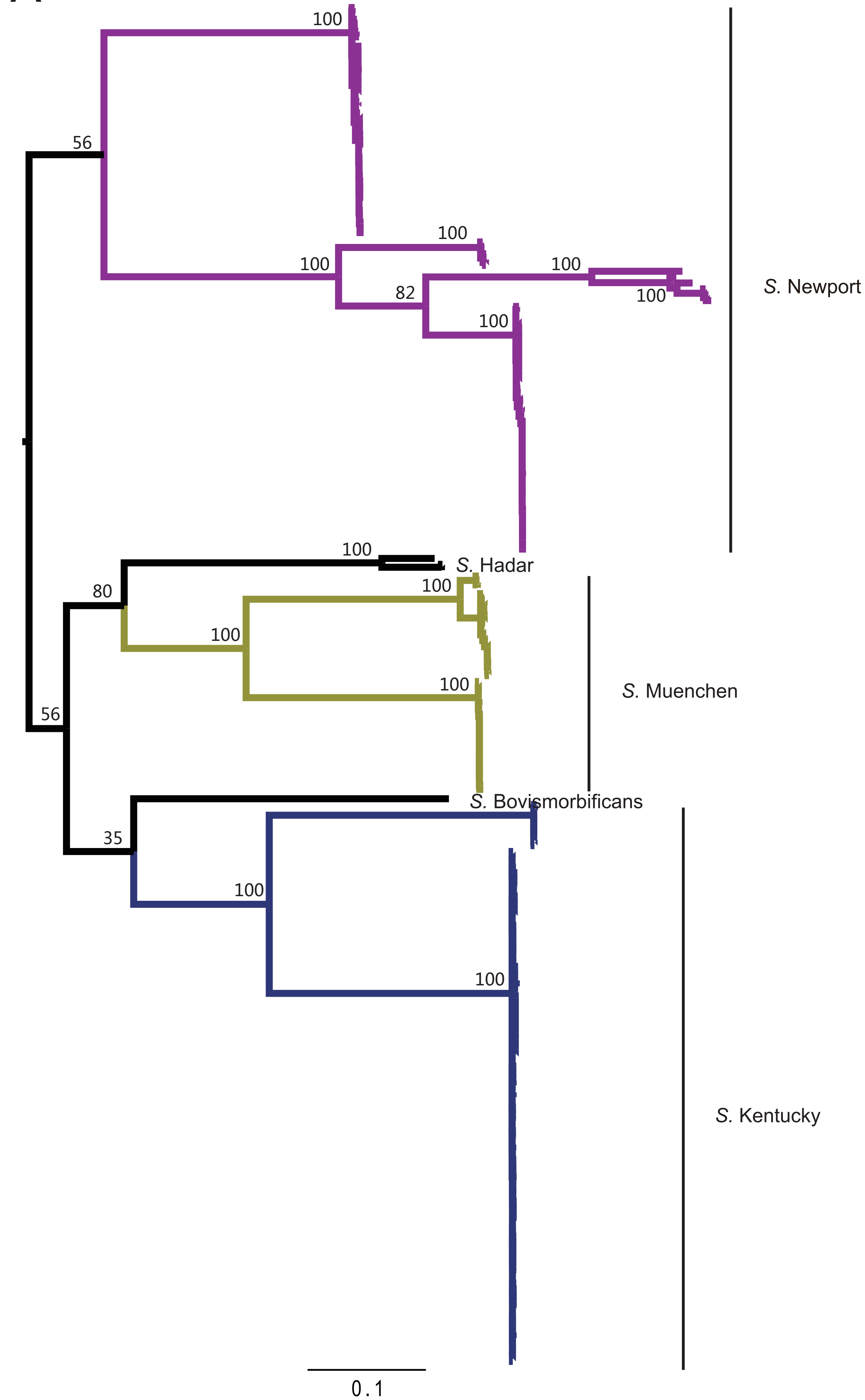

B H1 antigen tree

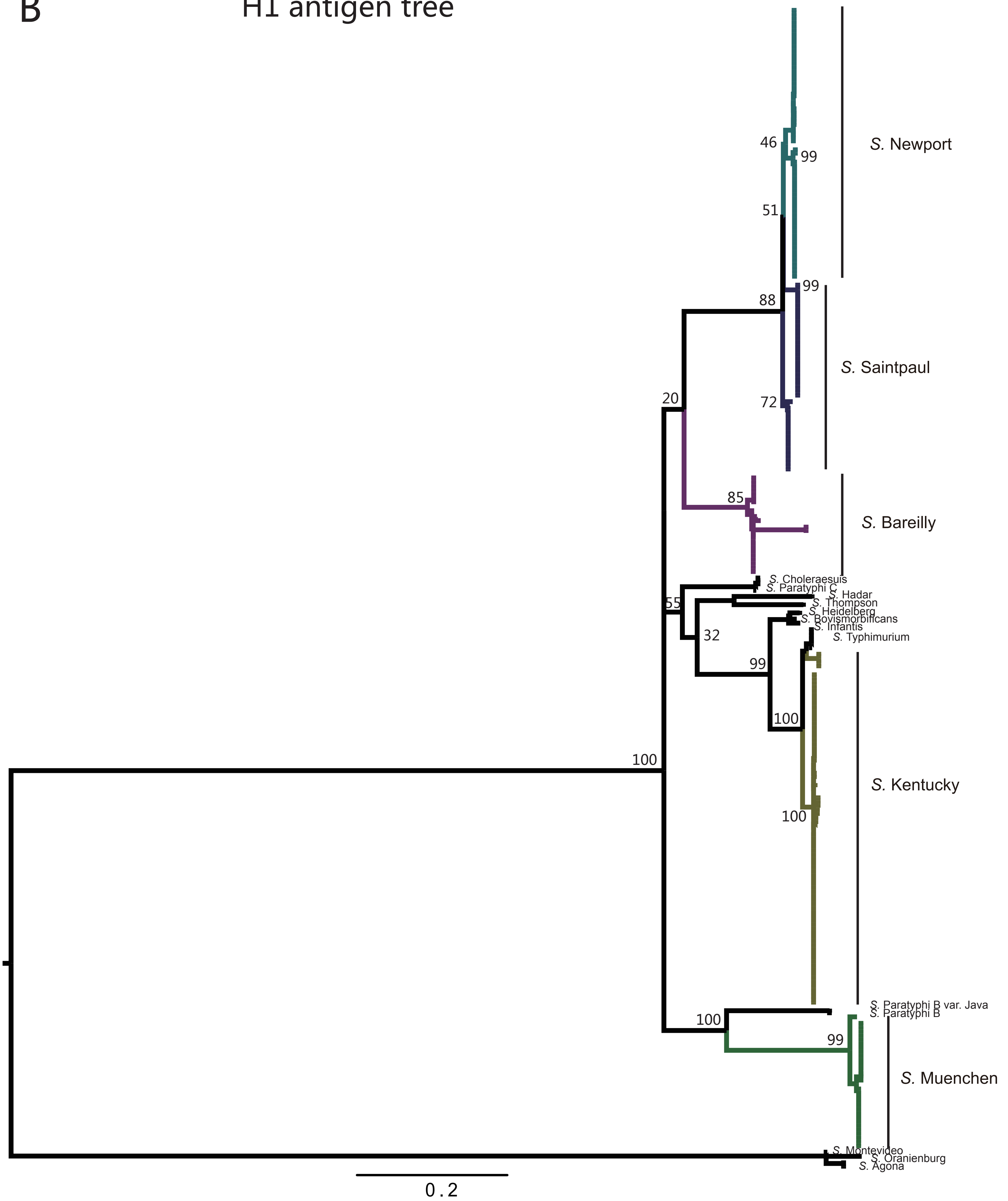

Supplement: Supplementary file 1 [file ijms-21-05226-s001.zip › Supplementary Files/Supplementary Figure S3.pdf]
